# A Bioreactor Technology for Modeling Fibrosis in Human and Rodent Precision‐Cut Liver Slices

**DOI:** 10.1002/hep.30651

**Published:** 2019-05-28

**Authors:** Hannah L. Paish, Lee H. Reed, Helen Brown, Mark C. Bryan, Olivier Govaere, Jack Leslie, Ben S. Barksby, Marina Garcia Macia, Abigail Watson, Xin Xu, Marco Y.W. Zaki, Laura Greaves, Julia Whitehall, Jeremy French, Steven A. White, Derek M. Manas, Stuart M. Robinson, Gabriele Spoletini, Clive Griffiths, Derek A. Mann, Lee A. Borthwick, Michael J. Drinnan, Jelena Mann, Fiona Oakley

**Affiliations:** ^1^ Newcastle Fibrosis Research Group, Institute of Cellular Medicine, Faculty of Medical Sciences Newcastle University Newcastle upon Tyne United Kingdom; ^2^ Liver Research Group, Institute of Cellular Medicine, Faculty of Medical Sciences Newcastle University Newcastle upon Tyne United Kingdom; ^3^ Newcastle University LLHW Centre for Ageing and Vitality Newcastle University Newcastle upon Tyne United Kingdom; ^4^ Wellcome Centre for Mitochondrial Research, Institute of Neuroscience Newcastle University Newcastle upon Tyne United Kingdom; ^5^ Department of Hepatobiliary Surgery Newcastle upon Tyne Hospitals NHS Foundation Trust Newcastle upon Tyne United Kingdom

## Abstract

Precision cut liver slices (PCLSs) retain the structure and cellular composition of the native liver and represent an improved system to study liver fibrosis compared to two‐dimensional mono‐ or co‐cultures. The aim of this study was to develop a bioreactor system to increase the healthy life span of PCLSs and model fibrogenesis. PCLSs were generated from normal rat or human liver, or fibrotic rat liver, and cultured in our bioreactor. PCLS function was quantified by albumin enzyme‐linked immunosorbent assay (ELISA). Fibrosis was induced in PCLSs by transforming growth factor beta 1 (TGFβ1) and platelet‐derived growth factor (PDGFββ) stimulation ± therapy. Fibrosis was assessed by gene expression, picrosirius red, and α‐smooth muscle actin staining, hydroxyproline assay, and soluble ELISAs. Bioreactor‐cultured PCLSs are viable, maintaining tissue structure, metabolic activity, and stable albumin secretion for up to 6 days under normoxic culture conditions. Conversely, standard static transwell‐cultured PCLSs rapidly deteriorate, and albumin secretion is significantly impaired by 48 hours. TGFβ1/PDGFββ stimulation of rat or human PCLSs induced fibrogenic gene expression, release of extracellular matrix proteins, activation of hepatic myofibroblasts, and histological fibrosis. Fibrogenesis slowly progresses over 6 days in cultured fibrotic rat PCLSs without exogenous challenge. Activin receptor‐like kinase 5 (Alk5) inhibitor (Alk5i), nintedanib, and obeticholic acid therapy limited fibrogenesis in TGFβ1/PDGFββ‐stimulated PCLSs, and Alk5i blunted progression of fibrosis in fibrotic PCLS. *Conclusion:* We describe a bioreactor technology that maintains functional PCLS cultures for 6 days. Bioreactor‐cultured PCLSs can be successfully used to model fibrogenesis and demonstrate efficacy of antifibrotic therapies.

**Abbreviations**
2D: two‐dimensional
3D: three‐dimensional
ALK5: activin receptor‐like kinase 5
ALK5i: Alk5 inhibitor
AST: aspartate aminotransferase
α‐SMA: α‐smooth muscle actin
CK19: cytokeratin 19
COL1a1: collagen 1a1
fPCLSs: fibrotic precision‐cut liver slices
ECM: extracellular matrix
ELISA: enzyme‐linked immunosorbent assay
H&E: hematoxylin and eosin
HM: hepatic myofibroblast
hPCLSs: human precision cut liver slices
HA: hyaluronic acid
IL: interleukin
LD: liver disease
MMP1: matrix metalloproteinase 1
MMP7: matrix metalloproteinase 7
MMP10: matrix metalloproteinase 10
NAFLD: nonalcoholic fatty liver disease
OCA: obeticolic acid
PCLSs: precision‐cut liver slices
PDGFββ: platelet‐derived growth factor
qHSCs: quiescent hepatic stellate cells
rPCLCs: rat PCLSs
TGFβ1: transforming growth factor beta 1
TIMP1: tissue inhibitor of metalloproteinase 1
VEGF: vascular endothelial growth factor

Hepatic fibrosis is characterized by accumulation of scar matrix in the liver and is the pathological consequence of persistent liver injury. Epithelial damage initiates local inflammation and activation of hepatic myofibroblasts (HMs), which secrete extracellular matrix (ECM) proteins to form a temporary scar. If the injury ceases, the scar is remodeled; however, persistent damage causes fibrosis.^1^

Currently, mono‐ or co‐cultures of liver cells, sandwich cultures, organoids, or animal models are used to interrogate the mechanisms driving the pathogenesis or reversion of liver disease and fibrosis to identify new targetable pathways and test antifibrotic drugs.^2^, ^3^, ^4^ These preclinical tools have strengths, but also limitations. Two‐dimensional (2D) cell cultures lack the physiologically relevant cell/cell and cell/ECM interactions found in the intact liver and are exposed to supraphysiological levels of mechanical stress when cultured directly on plastic. The latter drastically alters cell behavior; for example quiescent hepatic stellate cells (qHSCs) grown on rigid tissue culture plastic transdifferentiate into α‐smooth muscle actin (αSMA)^+^ HMs that express profibrotic genes and secrete ECM, whereas culturing qHSCs in soft Matrigel retains the features of HSC quiescence.^5^ Conversely, culturing HMs on soft matrix hydrogels (~2 kilopascals) supresses profibrotic gene expression and promotes a qHSC morphology.^5^, ^6^ Hepatocytes rapidly dedifferentiate and down‐regulate synthesis of albumin, metabolic enzymes, and cytochrome P450 after 24 hours in culture.^7^ Three‐dimensional (3D) spheroids provide an alternative system to grow hepatocytes or mixed liver cell cultures, but they fail to recapitulate the structural organisation of the liver or maintain physiologically relevant interactions with the ECM.^4^

Numerous animal models have been described utilizing different injurious stimuli to induce liver disease (LD)^3^ and study mechanisms of fibrosis. These models are mainly performed in young rodents, the disease inducer is often nonphysiological, and onset is rapid; there are metabolic differences between rodent and human liver meaning animals develop some, but not all, clinical features of the disease.^3^, ^8^

Precision cut liver slices (PCLSs) are used as an *ex vivo* culture model to study hepatic drug metabolism and fibrosis and benefit from retaining the 3D structure, physiological ECM composition, and complex cell/cell interactions of the liver.^9^, ^10^, ^11^ However, PCLSs cultured under static conditions in normoxia typically have a limited functional life span of ~24‐48 hours attributed to hypoxia. This causes death and disruption of tissue architecture and dramatically reduces secretion of the functional marker, albumin. Strategies to minimize the detrimental effects of hypoxia in PCLSs and limit accumulation of metabolites include: increasing oxygen concentration from 20% to 40%‐95%, using synthetic oxygen carriers (e.g., perfluorodecalin; varying media supplements/composition or introducing media flow by rocking or shaking the PCLS, using rotating culture vessels/rollers or perfusion circuits.^10^, ^12^, ^13^ Recently, an air‐liquid interface culture system has been used to model inflammation and immunological processes in human PCLSs over a 15‐day culture period, although of note this methodology was associated with significant spontaneous fibrogenesis and apoptosis.^14^ A significant advantage of PCLSs over animal models is that PCLSs can be generated from human liver; therefore, liver disease biology, efficacy of therapies, and drug metabolism can be modeled in human tissue *ex vivo*.^9^, ^15^, ^16^

We describe a bioreactor technology that maintains functional rodent and human PCLSs while minimizing tissue stress to enable inducible *ex vivo* modeling of induced fibrogenesis and preclinical studies examining the efficacy of antifibrotic interventions.

## Materials and Methods

### Animals

Ten‐ to 14‐week‐old Male Sprague‐Dawley rats housed in RC2 cages or 12‐week‐old male C57/Bl6 mice housed in individually ventilated cages were used. Cardboard tubes, bedding, and chew sticks provided environmental enrichment; animals had free access to RM3 diet (DBM diets, Broxburn, UK) and water and were maintained on a 12‐hour light/dark cycle. CCl_4_‐treated rats received a 1:3 mixture of CCl_4_ and olive oil (0.1 mL/100 g body weight by intraperitoneal injection) biweekly for 4 weeks to induce liver fibrosis (LF). Animals received humane care; experiments were approved by the Newcastle Animal Welfare and Ethical Review Board and performed under a UK Home Office license.

### Human Liver Tissue

Human liver tissue was obtained from normal resection margin surrounding colorectal metastasis, with informed consent from adult patients undergoing surgical resection at the Freeman Hospital (Newcastle‐upon‐Tyne, UK). This study was approved by the Newcastle & North Tyneside Research Ethics Committee (REC reference 12/NE/0395). Hepatocyte viability in culture is significantly reduced when isolated from livers >3 hours posthepatectomy.^17^ To minimize ischemic time and preserve hepatocyte viability, the maximum time for generating PCLS for experiments was 2 hours posthepatectomy.

### PCLSs

**Figure 1:**
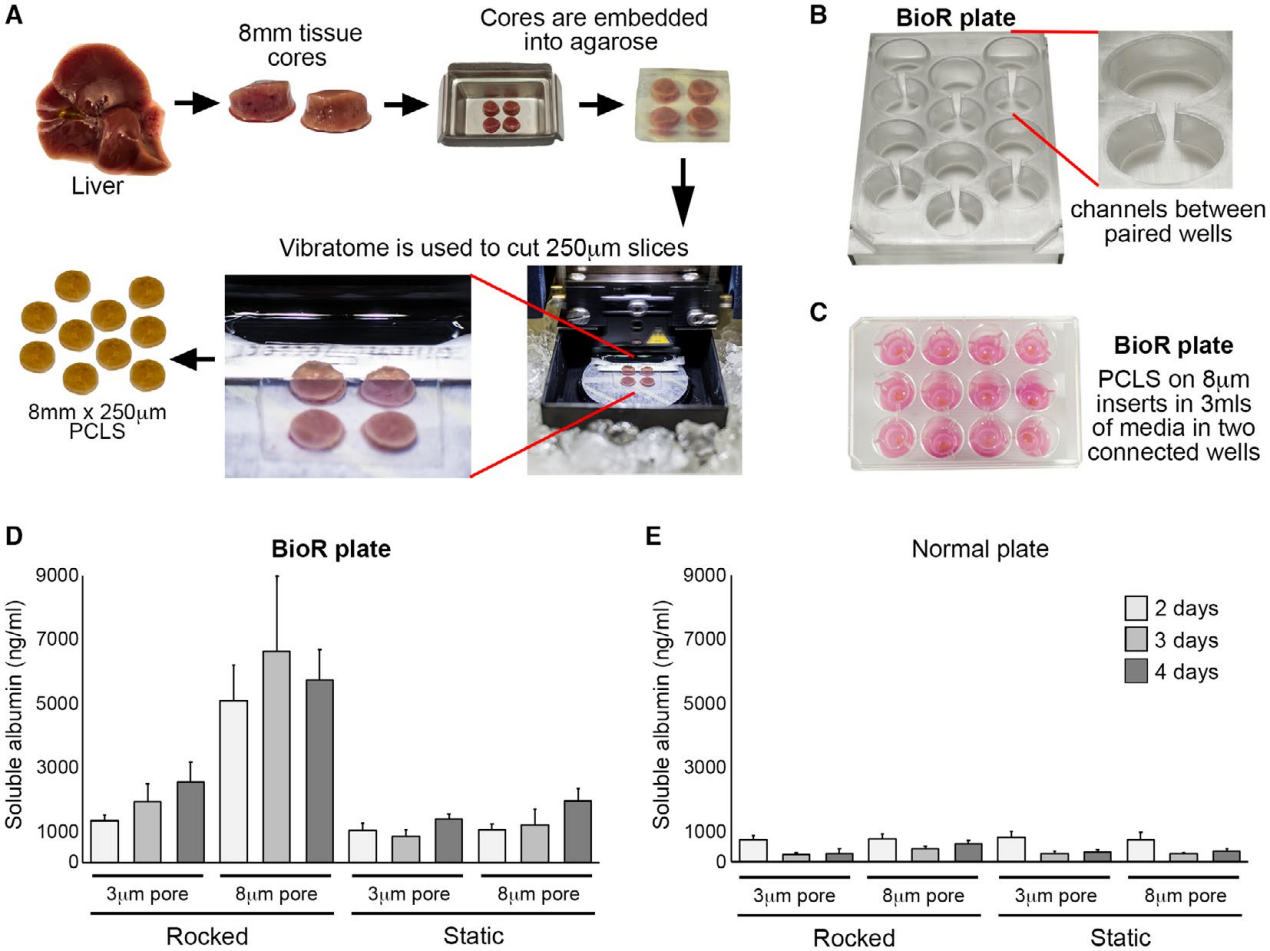
Generation of PCLSs and optimization of the bioreactor culture method. (A) Workflow used to generate PCLSs. (B) Photographs show the BioR plate containing six bioreactor chamber units and a zoomed image of one chamber unit, which contains two wells connected by a channel. (C) Photograph of PCLSs cultured on 8‐μm Transwell inserts in the BioR plate. (D,E) Graphs show secreted albumin (ng/mL) levels in media of PCLSs cultured on 3‐ or 8‐μm Transwell inserts in either a rocking BioR plate (D) or static 12‐well Transwell plate (E).

Liver tissue was cored using a 8‐mm Stiefel biopsy punch (Medisave, Weymouth, UK). Cores were transferred to a metal mould, submerged in 3% low geling temperature agarose (A9414; Sigma‐Aldrich, Poole, UK), and placed on ice for 2‐5 minutes. Agarose‐embedded liver cores were superglued to the vibratome mounting stage, submersed in the media chamber containing 4°C Hank’s balanced salt solution^+^, and cut using a Leica VT1200S vibrating blade microtome (Leica Biosystems, Milton Keynes, UK) at a speed 0.3 mm/sec, amplitude 2 mm, and thickness (step size) of 250 μm (Fig. 1A). PCLSs were transferred onto 8‐µm‐pore Transwell inserts and cultured under static conditions in standard 12‐well plates (Greiner Bio‐One, Kremsmuenster, Austria) or in a modified tissue culture plate (BioR plate) and rocked on the bioreactor platform (patent PCT/GB2016/053310) at a flow rate of 18.136 µL/sec (Fig. 1B,C and Supporting Fig. S1A). For comparison, PCLS were cultured in Kirstall Quasivivo (qv500) units according to the manufacturer’s instructions (Supporting Fig. S1B). All PCLSs were cultured in Williams medium E (W4128; Sigma‐Aldrich), supplemented with 1% penicillin/streptomycin and l‐glutamine, 1× insulin transferrin‐selenium X, and 2% fetal bovine serum (Thermo Fisher Scientific, Cramlington, UK), and 100 nM of dexamethasome (Cerilliant, Texas, USA) at 37°C, supplemented with 5% CO_2_. Media were changed daily.

### Modeling Fibrosis and Antifibrotic Drug Therapy

PCLSs were rested in culture for 24 hours, then stimulated ± 3 ng/mL of transforming growth factor beta 1 (TGFβ1) and 50 ng/mL of platelet‐derived growth factor (PDGFββ; PeproTech, London, UK) for a further 72 hours.

#### Therapy

PCLS were treated with 10 μM of activin receptor‐like kinase 5 (Alk5) inhibitor (Alk5i) SB‐525334, 2.5 μM of nintedanib, 10 μM of obeticholic acid (OCA), or 10 μM of losartan for 1 hour before fib stim and continued to be treated for the duration of experiment. Supernatants were collected at 24‐hourly intervals, and PCLSs were harvested for histological and biochemical assays.

Histological, biochemical methods, and statistical analysis are in the Supporting Information.

## Results

### The Bioreactor Extends the Life Span of Functional PCLSs

Currently, healthy life span of static PCLSs in normoxia is reported to be ~48 hours attributed to rapid tissue degradation and loss of function.^18^ To determine the optimal *in vitro* conditions for prolonged healthy maintenance of PCLSs, we assessed a number of parameters. To perform the tests, liver tissue was cored, embedded in agarose, and 8‐mm‐diameter, 250‐μm‐thickness PCLSs were prepared (Fig. 1A) and cultured under different conditions. Albumin production was used as an indicator of PCLS hepatocellular health and function. Initial pilot experiments demonstrated a need for continuous flow within PCLS cultures. Therefore, we developed a culture system consisting of a rocker and a BioR plate (Fig. 1B,C and Supporting Fig. S1A), which houses six bioreactor units, each unit comprising two cell‐culture wells connected by a channel (Fig. 1B, right panel). When the BioR plate is rocked on the bioreactor platform (Supporting Fig. S1A), media exchanges though the channel creating a bidirectional flow that helps oxygenate the slice and prevent accumulation of toxic metabolites. Slices were cultured on Transwell supports to prevent direct contact with plastic (Fig. 1C). Transwell pore size was crucially important for PCLS viability, with a pore size of 8 μm preserving stable albumin secretion over 4 days (Fig. 1D). Conversely, albumin production rapidly decreased in PCLSs cultured on 3‐μm‐pore Transwell inserts in either a static BioR plate (i.e., without rocking) or a rocked BioR plate (Fig. 1D). By comparison, albumin was significantly attenuated in PCLSs cultured on 3‐ or 8‐μm Transwell inserts in standard 12‐well plates (without a connecting channel between wells) under static conditions (Fig. 1E). Importantly, rocking standard 12‐well plates did not improve hepatocellular function of PCLSs, even when cultured on 8‐μm Transwell inserts (Fig. 1E). We next asked whether bidirectional flow was important and compared PCLSs cultured in rocked BioR plates exposed to bidirectional flow, with PCLSs cultured under unidirectional flow, in a Quasi‐Vivo circuit (Supporting Fig. S1B). As expected, albumin production remained stable over 4 days in the bioreactor PCLSs, but decreased by ~90% between days 2 and 3 under unidirectional flow (Supporting Fig. S1B).

We next evaluated the histological appearance of PCLSs cultured in the bioreactor or static Transwell plates. Static‐cultured PCLSs showed tissue disruption/degradation as early as 2 days, with loss of endothelial cells, atrophy of bile ducts, sinusoidal dilatation, and loss of hepatocyte nuclei from days 2 to 6 in culture (Supporting Fig. S2A). Conversely, bioreactor‐cultured PCLSs retained structural integrity and the morphology of the portal tract and parenchyma (Supporting Fig. S2A). Consistent with the improved histological appearance of bioreactor‐cultured PCLSs, liver albumin production was stable over the 6‐day culture period whereas albumin levels were 33% lower in static‐cultured PCLSs after 24 hours and significantly reduced over the next 5 days, reaching only 20%‐25% of the time‐matched, bioreactor‐cultured PCLSs (Supporting Fig. S2B). Seahorse analysis and Resazurin assays confirmed that bioreactor‐cultured PCLSs remained metabolically active in culture, whereas the viability and metabolic activity of static‐cultured PCLSs was significantly reduced as early as 24 hours (Supporting Fig. S2C,D). Collectively, these data confirm that culturing PCLSs in our bioreactor under normoxic culture conditions and in the absence of oxygen carriers significantly increases the life span of functional PCLSs compared to static Transwell cultures. All future experiments were therefore conducted on PCLSs cultured in the bioreactor.

**Figure 2:**
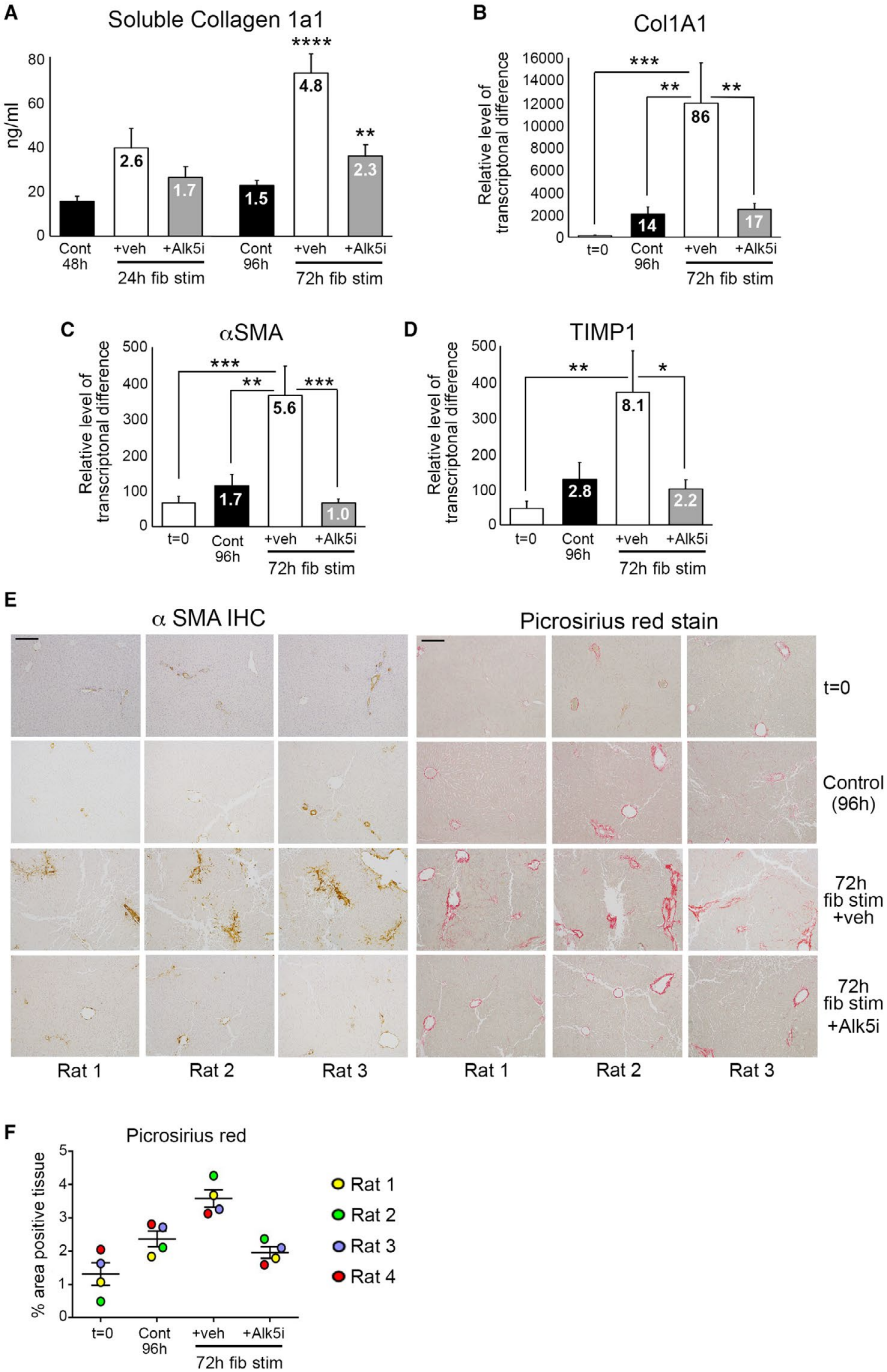
Modeling fibrosis and testing antifibrotic therapy in normal rat liver slices. (A) Graph shows soluble Col1a1 (ng/mL) levels in media of bioreactor‐cultured rPCLSs after 24‐hour rest and then 24‐ and 72‐hour culture ± fib stim (TGFβ1/PDGFββ) ± Alk5i (48‐ and 96‐hour total culture). (B‐D) mRNA levels of Col1A1, αSMA, and TIMP1 in rPCLSs at t = 0 and after 24‐hour rest + 72‐hour culture ± fib stim ± Alk5i (96‐hour total culture). (A‐D) Numbers on bars show fold change compared to t = 0. (E) Representative 100× magnification images of αSMA and picrosirius red–stained rPCLSs from three different livers at t = 0 and after 24‐hour rest + 72‐hour culture ± fib stim ± Alk5i. Scale bars = 200 µm. (F) Graph shows percentage area of picrosirius red–stained tissue in rPCLS at t = 0 and after 24‐hour rest + 72‐hour culture ± fib stim ± Alk5i. Data are mean ± SEM in n = 4 different livers. *P < 0.05; **P < 0.01; ***P < 0.001; ***P < 0.0001. Abbreviations: Cont, control; fib stim, fibrotic stimulation; IHC, immunohistochemistry; Veh, vehicle.

The increased longevity of bioreactor‐cultured PCLSs that retain cellular composition and tissue architecture provides the opportunity to induce fibrosis in PCLSs and assess efficacy of antifibrotic drugs. PCLSs were generated from healthy rat liver, rested for 24 hours, and then stimulated for up to 72 hours (up to 96‐hour total culture period) with or without the profibrotic stimuli TGFβ1/PDGFββ, which stimulate HM proliferation and survival. After 96‐hour culture, basal soluble collagen 1a1 (Col1a1) secretion from control rat PCLSs (rPCLSs) into culture media increased 1.5‐fold, compared to 48‐hour control slices (24 hours rest + 24 hours no treatment; Fig. 2A). Conversely, Col1a1 secretion increased 2.6‐fold after 24 hours and 4.8‐fold after 72‐hour TGFβ1/PDGFββ stimulation, which reached significance at 72 hours posttreatment (Fig. 2A). Col1a1 secretion was significantly attenuated by an Alk5i, a potent suppressor of TGFβ type I receptor‐dependant signaling.^19^ Expression of profibrotic genes Col1a1, αSMA, and tissue inhibitor of metalloproteinase 1 (TIMP1) were significantly elevated after 72 hours of TGFβ1/PDGFββ stimulation, and this was supressed by Alk5i (Fig. 2B‐D). Whereas αSMA and TIMP1 levels in 96‐hour control PCLSs were modestly increased compared to t = 0 normal liver, collagen gene expression was significantly increased (14‐fold) in control PCLSs compared to t = 0. However, important to note was that the increased collagen gene expression did not translate to increased secretion of Col1a1 or deposition of fibrotic matrix within PCLSs (Fig. 2A,E).

We next confirmed histologically that TGFβ1/PDGFββ promoted deposition of fibrotic matrix in PCLSs (Fig. 2E). In t = 0 healthy rat PCLSs, αSMA and picrosirius red staining was restricted to vessels. Occasional αSMA^+^ HMs were present in 4‐day–cultured control PCLSs, whereas picrosirius red staining showed thickening of collagen around central veins and modest perisinusoidal collagen deposition (Fig. 2E). Conversely, TGFβ1/PDGFββ induced activation of HMs and more‐extensive perisinusoidal fibrosis, with thick multilayered collagen present around vessels. Alk5i blunted these histological changes, such that αSMA and picrosirius red staining was similar between control and TGFβ1/PDGFββ + Alk5i PCLS (Fig. 2E). Collagen deposition, assessed by picrosirius red area analysis, showed a small increase in collagen area in 4‐day control PCLSs compared to the donor matched t = 0 PCLSs. TGFβ1/PDGFββ stimulation significantly increased collagen deposition in all donors compared to t = 0 and control cultured PCLSs, and this was significantly attenuated by Alk5i therapy (Fig. 2F). Importantly, albumin, urea, and aspartate aminotransferase (AST) release by PCLSs were not significantly different between the various experimental conditions, suggesting that fibrotic stimuli or Alk5i therapy did not cause liver toxicity or effect hepatocellular function (Supporting Fig. S3A‐C).

**Figure 3:**
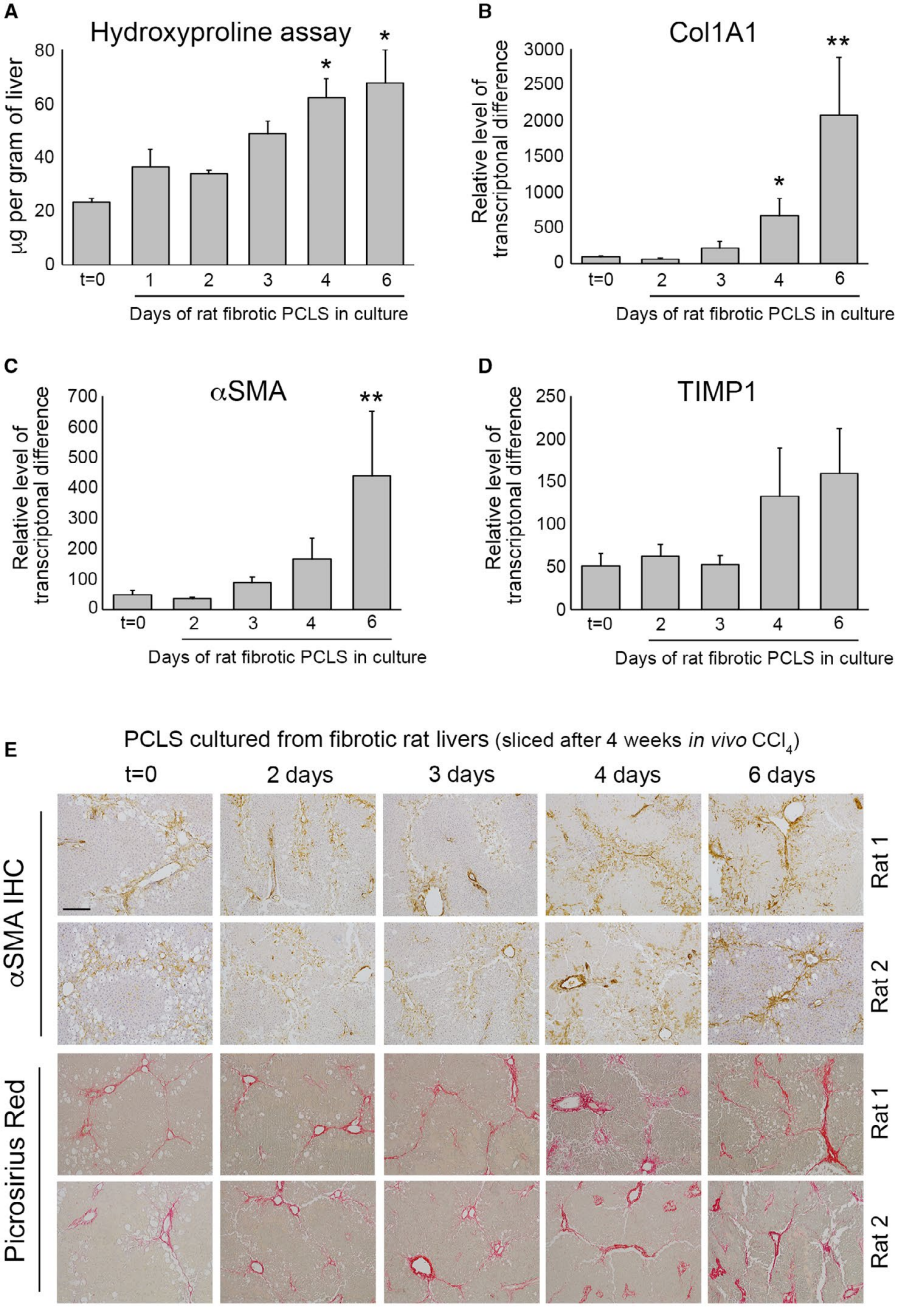
Bioreactor‐cultured fibrotic rat liver slices can be maintained for 6 days. (A) Graph shows hydroxyproline (μg/g of liver tissue) in bioreactor‐cultured fPCLSs. (B‐D) mRNA expression of Col1A1, αSMA, and TIMP1 in bioreactor‐cultured fPCLSs at t = 0 and 2‐, 3‐, 4‐, and 6‐day culture. Data are mean ± SEM in n = 5 independent slice experiments. (E) Representative images of αSMA and picrosirius red–stained rat fPCLS at t = 0 and 2, 3, 4, and 6 days in culture. Scale bars = 200 µm. *P < 0.05; **P < 0.01. Abbreviation: IHC, immunohistochemistry.

We next asked whether fibrosis is stable or dynamic in bioreactor‐culture PCLSs using preestablished fibrotic PCLSs (fPCLSs), which were generated from 4‐week CCl_4_‐treated rats and cultured for up to 6 days. At t = 0, hematoxylin and eosin (H&E)‐stained PCLSs displayed the expected features of CCl_4_‐induced liver injury, including pericentral damage, tissue necrosis, and ballooned hepatocytes (Supporting Fig. S4A). Consistent with liver damage, AST levels were high after 24 hours in culture, but decreased by 48 hours and continued to steadily decrease up to 6 days (Supporting Fig. S4B). After a 24‐hour rest period, albumin production by fPCLSs was slightly reduced, but remained stable, from days 2 to 6 (Supporting Fig. S4C). Levels of hydroxyproline, a major component of fibrotic matrix, was stable in fPCLSs for 48 hours, but gradually increased with time, this effect reaching significance at 4 and 6 days, suggesting that ECM biosynthesis and deposition are progressive in cultured fPCLSs (Fig. 3A). This was accompanied by a significant increase in Col1a1 gene expression at days 4 and 6 (Fig. 3B). Similarly, expression of αSMA and TIMP1 increased at days 4 and 6 (Fig. 3C,D). Histologically, t = 0 fPCLSs revealed damaged hepatocytes, a characteristic “chicken wire” distribution of αSMA^+^ HMs surrounding the central veins and bridging fibrosis (Fig. 3E). Consistent with the biochemical data, distribution of HM and bridging fibrosis is stable for 3 days; however HM numbers and picrosirius red–stained area increase by 4‐6 days in culture. Picrosirius red staining revealed a thickening of fibrous septa, increased intensity of collagen staining, presence of multilayered collagen, and periportal fibrosis at 4‐6 days (Fig. 3E). These data confirm that fPCLSs are a dynamic system in which an active process of progressive net fibrogenesis occurs. We therefore asked whether Alk5i could suppress fibrogenesis in fPCLSs. Hydroxyproline concentration was increased 2‐fold in 4‐day–cultured fPCLSs compared to t = 0, and this was significantly attenuated by Alk5i treatment (Supporting Fig. S4D).

**Figure 4:**
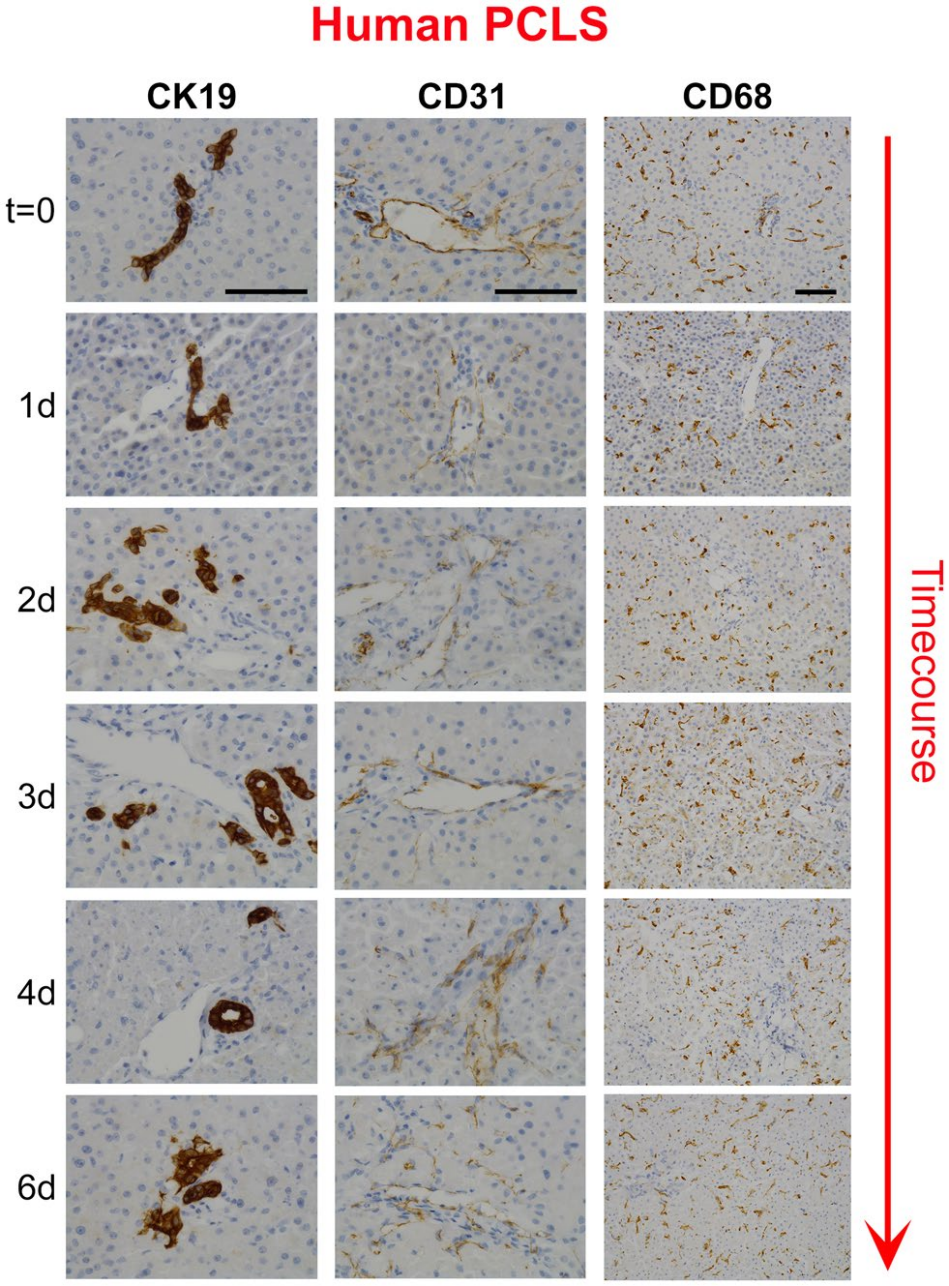
Bioreactor‐cultured human liver slices retain ductular cells, endothelial cells, and macrophages. Representative images show CK19‐stained biliary epithelial cells (left), CD31‐stained endothelial cells (middle), and CD68‐stained macrophages (right) in t = 0 and days 1, 2, 3, 4, and 6 cultured hPCLS. CK19 and CD31 images are at 400× magnification and CD68 are at 200× magnification.

**Figure 5:**
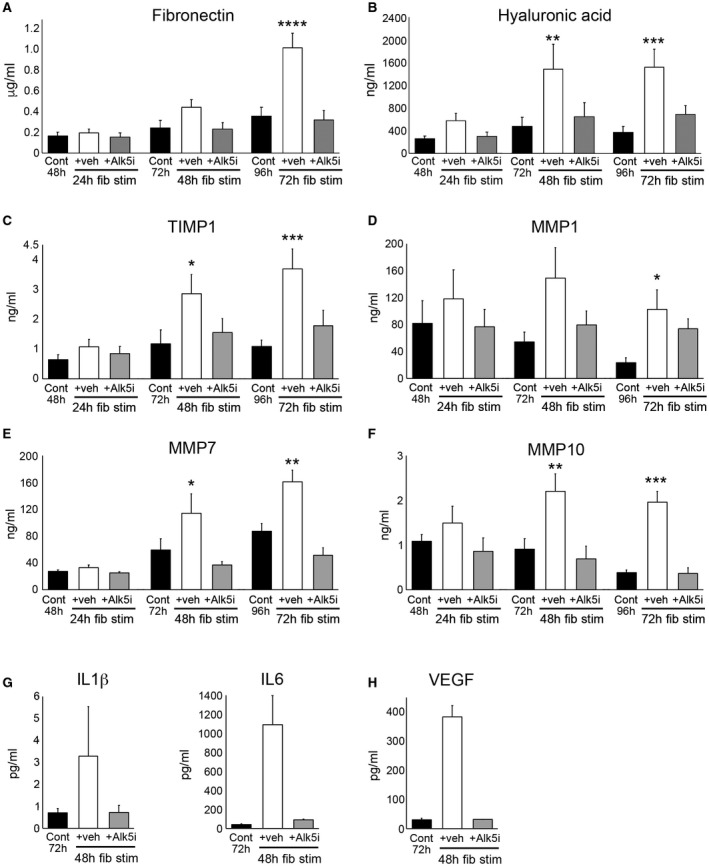
Modeling fibrosis and testing antifibrotic therapy in human liver slices using soluble markers. (A‐H) Graphs show fibronectin (μg/mL), HA (ng/mL), TIMP1 (ng/mL), MMP1 (ng/mL), MMP7 (ng/mL), MMP10 (ng/mL), IL‐6 (pg/mL), IL‐1β (pg/mL), and VEGF (pg/mL) levels in media of bioreactor‐cultured hPCLSs after 24‐hour rest and then 24‐ or 72‐hour culture ± fib stim (TGFβ1/PDGFββ) ± Alk5i; numbers on bars show fold change compared to 48‐hour control. Data are mean ± SEM in n = 4 independent slice experiments. *P < 0.05; **P < 0.01; ***P < 0.001; ****P < 0.0001. Abbreviations: Cont, control; fib stim, fibrotic stimulation; Veh, vehicle.

**Figure 6:**
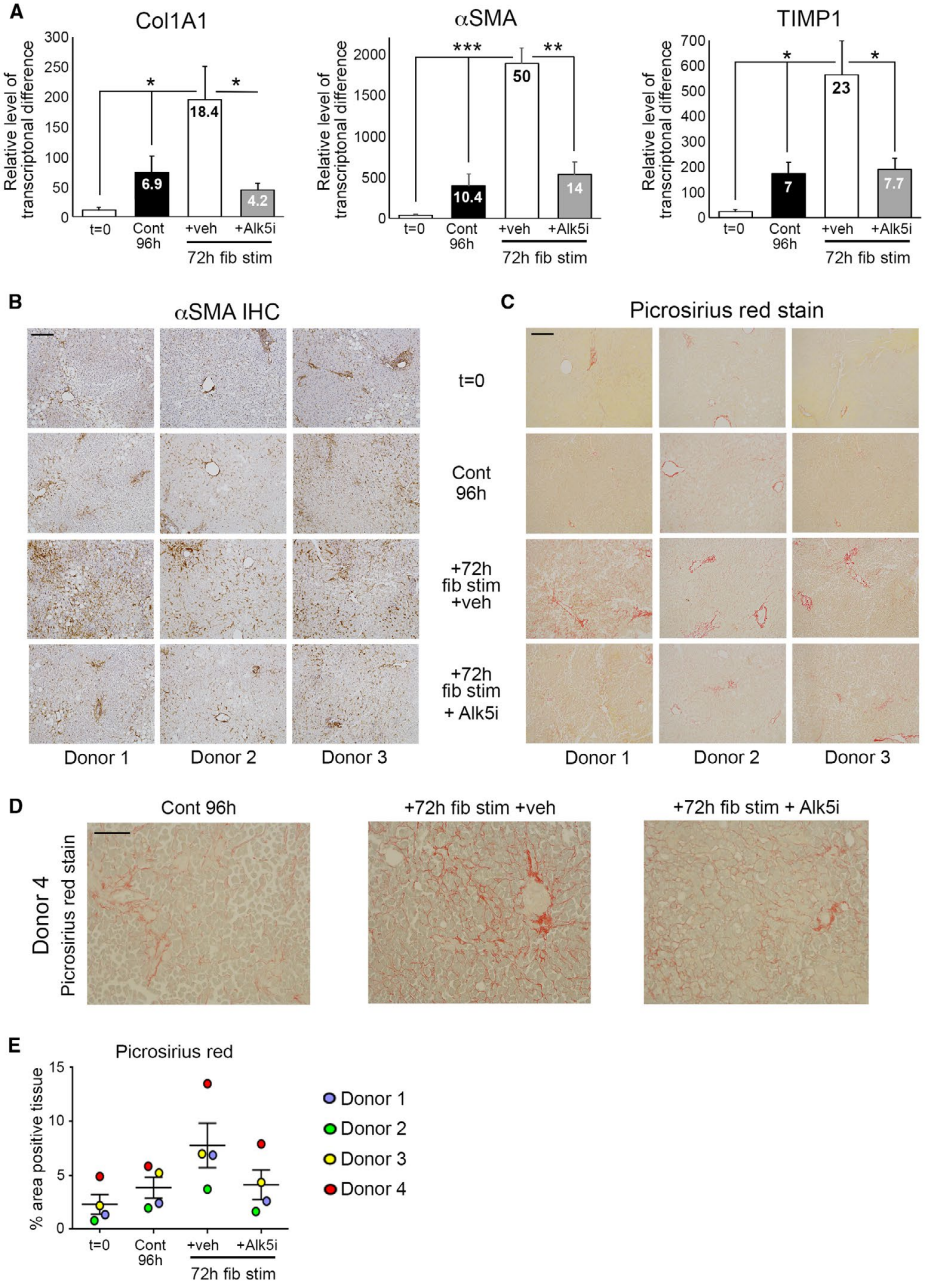
Modeling fibrosis and testing antifibrotic therapy in human liver slices using gene expression and histology. (A) mRNA levels of Col1A1, αSMA, and TIMP1 in hPCLS at t = 0 and after 24‐hour rest + 72‐hour culture ± fib stim ± Alk5i; numbers on bars show fold change compared to t = 0. (B) Representative 100× magnification images of αSMA and (C) picrosirius red–stained hPCLSs from three different donor livers at t = 0 and after 24‐h rest + 72‐hour culture (4 days) ± fib stim ± Alk5i. Scale bars = 200 µm. (D) Representative 200× magnification image of picrosirius red–stained hPCLSs from donor 4. (E) Percentage area of picrosirius red–stained tissue in hPCLSs at t = 0 and after 24‐hour rest + 72‐hour culture ± fib stim ± Alk5i (96‐hour total culture). Data are mean ± SEM in n = 4 independent slice experiments. *P < 0.05; **P < 0.01; ***P < 0.001; ****P < 0.0001. Abbreviations: Cont, control; fib stim, fibrotic stimulation; IHC, immunohistochemistry; Veh, vehicle.

An advantage of PCLSs is to model fibrogenesis and assess antifibrotic therapies in human liver tissue. Histological characterization of bioreactor‐cultured human PCLSs (hPCLSs) confirmed that tissue structure and expression of cytokeratin 19 (CK19)^+^ biliary epithelial cells, CD31^+^ endothelial cells, CD68^+^ macrophages, the cytochrome P450 enzyme, cytochrome P450 family 1 subfamily A member 2, mitochondrial respiratory chain complexes I (NADH dehydrogenase [ubiquinone] 1 beta subcomplex subunit 8) and IV (mitochondrially encoded cytochrome c oxidase I), which are lost in the presence of mitochondrial dysfunction,^20^ and the mitochondrial mass marker, translocase of outer mitochondrial membrane 20, are retained throughout a 6‐day culture period (Fig. 4 and Supporting Figs. S5 and S6). Therefore, we tested whether fibrogenesis can be induced in bioreactor‐cultured hPCLSs and whether an Alk5i limited this. hPCLSs were cultured for a 24‐hour “rest period” and then treated ± TGFβ1/PDGFββ ± Alk5i for up to 72 hours (96‐hour total culture). In the absence of fibrotic stimuli, culturing PCLSs for 96 hours induced a small increase in secretion of the soluble ECM components, fibronectin (2.2‐fold) and hyaluronic acid (HA; 1.4‐fold), into culture media compared to 48‐hour control PCLSs (Fig. 5A,B). Conversely, 72 hour TGFβ1/PDGFββ treatment significantly increased fibronectin (6.2‐fold) and HA (5.9‐fold) secretion compared to control PCLSs (Fig. 5A,B). Similarly, secretion of matrix regulators TIMP1, matrix metalloproteinase 1 (MMP1), matrix metalloproteinase 7 (MMP7), and matrix metalloproteinase 10 (MMP10) were significantly elevated after 48‐hour TGFβ1/PDGFββ stimulation and by 72‐hour TGFβ1/PDGFββ stimulation; TIMP1 (5.8‐fold), MMP1 (1.3‐fold), MMP7 (5.9‐fold), and MMP10 (1.8‐fold; Fig. 5C‐F). Release of soluble inflammatory mediators interleukin (IL)‐6 and IL‐1β and the proangiogenic protein, vascular endothelial growth factor (VEGF), were also increased in TGFβ1/PDGFββ‐challenged PCLSs (Fig. 5G,H), highlighting that multiple features of wound healing are inducible in our PCLS system. Importantly, secretion of ECM proteins was accompanied by an elevation in Col1A1, αSMA, and TIMP1 gene expression (Fig. 6A). Compared to t = 0, Col1A1, αSMA, and TIMP1 expression was increased by 18.4‐, 50‐, and 23‐fold respectively in TGFβ1/PDGFββ‐treated hPCLSs. Alk5i suppressed hPCLS secretion of ECM components, matrix regulators, and fibrosis gene expression (Figs. 5A‐F and 6A). It has been reported that in human liver, αSMA^+^ cells are present in the portal tracts, perivenular space, and liver lobule with discontinuous staining of qHSCs in adult human liver biopsies or surgical resections.^21^ Consistent with this report, αSMA^+^ cells were detected in central veins, portal tracts, and perisinusoidal spaces of normal donor liver (Supporting Fig. S7A). Picrosirius red–stained collagen was predominantly restricted to the vessels with delicate sinusoidal staining in nonsliced donor livers (Supporting Fig. S7B). The histological appearance of αSMA and picrosirius red in t = 0 postslice hPCLSs was consistent with the nonsliced human liver, suggesting that the preparation method and physical process of slicing did not promote acute HM activation or fibrotic changes (Fig. 6B‐D). Treatment of hPCLSs with fibrotic stimuli promoted accumulation of αSMA^+^ HMs throughout the parenchyma and adjacent to the central veins and portal tracts (Fig. 6B‐D). Early fibrotic changes in hPCLSs cultured with fibrotic stimuli were evident by the presence of more‐intense and ‐extensive perisinusoidal picrosirius red staining and thickening of collagen around the vessels. Fibrotic stimuli induced HM activation and collagen deposition was attenuated by an Alk5i, and the histological appearance was similar to 96‐hour control PCLS, showing a small increase in HM activation and modest persinusoidal collagen deposition compared to t = 0 (Fig. 6B‐D). Picrosirius red area analysis confirmed an insignificant, minor increase in collagen area in 4‐day control hPCLSs compared to donor matched t = 0 PCLSs. TGFβ1/PDGFββ stimulation further increased collagen area in all donors, and this was blunted and comparable to control levels after Alk5i therapy (Fig. 6E). Importantly, stimulation of hPCLS with TGFβ1/PDGFββ ± ALK5i did not significantly affect albumin or urea production (Supporting Fig. S8A,B).

**Figure 7:**
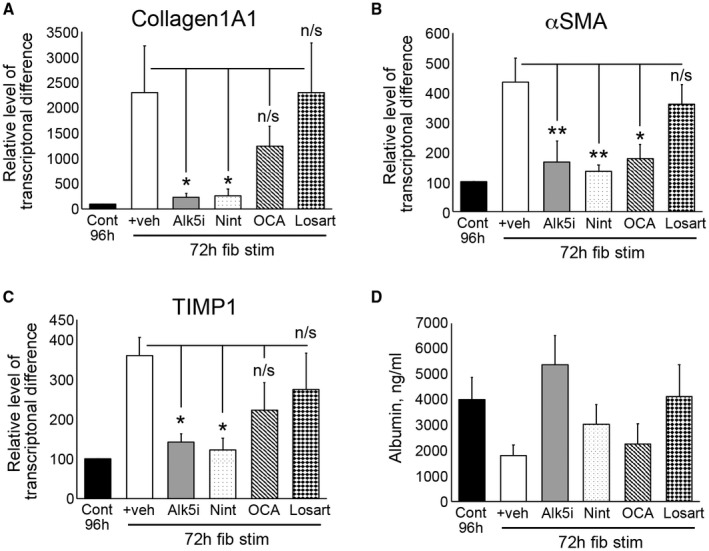
Assessing impact antifibrotic therapies in fibrosis‐induced rat liver slices. (A‐C) mRNA levels of Col1A1, αSMA, and TIMP1 in rPCLS at t = 0 and after 24‐hour rest + 72‐hour culture ± fib stim ± nintedanib, OCA, and losartan (96‐hour total culture). Data are mean ± SEM in n = 5 different livers. *P < 0.05; **P < 0.01; ***P < 0.001; ***P < 0.0001. (D) Average media albumin (ng/mL) levels in bioreactor‐cultured rPCLSs after 24‐hour rest and then 24‐ to 72‐hour culture ± fib stim (TGFβ1/PDGFββ) ± Alk5i, nintedanib, OCA, and losartan (48‐ and 96‐hour total culture). Abbreviations: Cont, control; fib stim, fibrotic stimulation; Nint, nintedanib; Veh, vehicle.

Finally, we asked whether fibrosis‐induced PCLSs could predict response of antifibrotic therapies. As expected, the Alk5i significantly reduced Col1A1, αSMA, and TIMP1 gene expression (Fig. 7A‐C). Nintedanib, a licensed therapy for idiopathic pulmonary fibrosis, significantly attenuated fibrogenic gene expression (Fig. 7A‐C). The hepatic protective drug, OCA, significantly reduced αSMA expression and modestly reduced TIMP1 levels; however, losartan, which has generated inconclusive results in clinical trials, had no effect on fibrotic gene expression (Fig. 7A‐C). None of the drugs significantly altered albumin production (Fig. 7D).

## Discussion

We describe a bioreactor that extends the health, metabolic activity, and functional longevity of PCLSs to at least 6 days and, importantly, without obvious hepatocellular stress and significant spontaneous fibrogenesis. Moreover, we report that active induced fibrogenesis can be modeled using TGFβ1/PDGFββ stimulation, and that an Alk5 inhibitor attenuates this process. Importantly, to evaluate fibrogenesis in our PCLSs, we have quantified multiple biological outputs, including secretion of ECM components, changes in fibrogenic gene expression, tissue collagen (hydroxyproline assay), and histological changes. Notably, the fibrogenic outputs can be reproducibly measured in PCLSs stimulated with TGFβ1/PDGFββ within a relatively short time frame (3 days), providing a rapid model for testing potential antifibrotic drugs in high‐quality, functional human liver tissue. Furthermore, the bioreactor supports extended culture of fibrotic liver that becomes self‐sustainable in culture, but remains modulatable using an Alk5i. Critically, the ability of antifibrotic compounds in clinical use/trials (Fig. 7) or drugs to block fibrogenesis in PCLSs can be compared to effective inhibition mediated by Alk5i.

Multiple published studies describe different methodologies to culture human and/or rodent PCLSs for modeling liver toxicity, investigating the effects of alcohol, studying fibrosis, or evaluating antifibrotic drug efficacy.^11^, ^22^, ^23^, ^24^ However, several critical differences between the PCLS culture conditions in other studies compared to our bioreactor are noteworthy. Previous studies using PCLSs cultured directly on plastic have a severely limited functional life span (<48 hours); however, this has been extended up to 72 hours by culturing the slice in high‐oxygen concentrations with or without gentle shaking in a circular motion.^22^, ^23^ Whereas most culture systems use fully submerged PCLSs, one study described a rocked platform where hPCLSs are cultured on organoid Transwell plates in an air‐liquid interface in normoxia.^14^ In the air‐liquid interface system, PCLSs are exposed to air on the upper side and are submerged in media on the lower side. This alternative system was used to evaluate immunological responses to viral and bacterial products in PCLSs at a gene expression level,^14^ but of note the investigators reported substantial fibrogenesis, which prevented the model from being used to study induced fibrosis. By contrast, our bioreactor was specifically designed to minimize hepatocellular damage to limit background fibrogenesis, thereby enabling investigations of induced fibrosis. Three key features help the bioreactor to maintain functionality and extend the life span of viable PCLSs. First, a combination of rocking and gravity promotes media exchange between the two adjacent wells of the bioreactor chamber, creating a bidirectional flow that helps minimize hypoxia and reduce accumulation of toxic metabolites within the PCLSs. Media flow has been closely matched to the sinusoidal flow within intact liver. Second, PCLSs are cultured on semipermeable Transwell inserts (8‐μm pores) that facilitate media exchange from above and below the slice, and prevent direct contact with stiff tissue culture plastic. Third, PCLSs are cultured under physiologically relevant normoxic conditions.

Our bioreactor uses gentle rocking to induce flow and therefore does not require a peristaltic pump. Whereas perfusion systems require multiple perfusion circuits to perform multiple treatments,^25^ our bioreactor is quick and easy to assemble, with scope to scale up to many concurrently running bioreactors for large studies with multiple experimental parameters or compound dose responses.

Our development of a hPCLS model of fibrosis is important given that it provides a robust platform with which to strengthen preclinical confidence in potential therapeutic targets/drugs for future clinical trials. For example, compounds demonstrating antifibrotic effects in rodent fibrosis models and 2D/3D cell‐culture platforms could be screened in hPCLSs for drug metabolism, toxicity, and efficacy in human tissue. Moreover, modeling fibrogenesis in PCLSs would reduce animal use in accord with the 3Rs; reduction, replacement, and refinement. Approximately 150‐200 PCLSs can be generated from a normal or diseased rat liver, providing a unique opportunity to perform medium‐throughput drug screening/dose determination experiments in fewer animals before *in vivo* studies. Compounds exhibiting therapeutic actions in 2D cultures and rodent PCLS would be progressed to hPCLSs for focused drug screening to gain important information about target engagement/efficacy in human liver tissue. These approaches could significantly reduce animal use and help replace moderate and severe *in vivo* procedures. Another advantage of hPCLSs is daily media sampling, which raises the possibility that clinically relevant soluble biomarkers can be measured longitudinally to monitor disease progression or assess drug efficacy. Here, we measured HA, TIMP1s and MMPs released from hPCLSs as surrogate markers of fibrogenesis, which have been reported as predictive biomarkers. Serum HA levels have been shown to correlate with fibrosis grades in hepatitis C patients,^26^ whilst in a separate hepatitis C patient cohort, MMP7 and TIMP1 was used to stratify patients without and with cirrhosis.^27^ In nonalcoholic fatty liver disease (NAFLD) patients, serum TIMP1 has diagnostic value in distinguishing patients with fibrosis,^28^ whereas another study correlated MMP1 levels with early fibrosis.^29^ Measuring serum markers in chronic LD patients without fibrosis versus those with advanced fibrosis revealed that HA, MMP7, and MMP1, which were all elevated in our fibrotic PCLSs, were an accurate predictor of fibrosis when combined with alpha‐fetoprotein, AST, and the AST/platelet ratio index.^30^ Collectively, our PCLS system mirrors the effectiveness of drugs tested in clinical trials and could represent a powerful tool for preclinical testing.

Although PCLSs have significant advantages over cell‐culture models, one limitation is the absence of infiltrating immune cells, which can modulate the disease process. A future development for our PCLS system will be to egress immune cells into normal or diseased PCLSs. This would provide an opportunity to investigate how different immune cell populations modulate disease biology and fibrogenesis in intact human tissue.

Currently, our bioreactor technology has been used to study TGFβ1/PDGFββ‐induced LF and test antifibrotic drugs; however, in the future, the utility of the system can be significantly increased by developing new disease models in hPCLSs that faithfully recreate the features of different etiologies of LD. Development of bespoke LD models in hPCLSs could be utilized to elucidate the disease‐specific mechanisms promoting disease progression, but also identify core mechanisms of disease pathogenesis and fibrosis. Treating PCLSs with disease‐relevant lipids and sugars could model NAFLD, whereas addition of lipids and bacterial products and/or immune cells could recreate features of advanced NAFLD and nonalcoholic steatohepatitis, whereas culturing PCLSs with ethanol or alcohol metabolites, either with or without immune cells, is a strategy to model alcoholic liver disease. The system also lends itself to assess drug metabolism and drug‐induced liver toxicity or model liver failure using acetaminophen. Currently, 2D hepatoceullar carcinoma cell‐line cultures, xenografts, or chemical‐induced animal models of liver cancer are used to identify drugable biological process/pathways. Addition of liver cancer cell lines or patient‐derived cancer cells to bioreactor‐cultured hPCLSs could represent an improved system to study cancer growth or test antitumor therapies within the context of a 3D liver environment.

Establishing improved preclinical models in our bioreactor‐cultured PCLSs to study liver disease and cancer would aid drug discovery and permit targeted testing of new therapeutics in clinically relevant disease models.
