# Supplementary material for: A Bioreactor Technology for Modeling Fibrosis in Human and Rodent Precision‐Cut Liver Slices
Source: Hepatology. 2019 May 28;70(4):1377–91. doi: 10.1002/hep.30651 (PMC6852483; doi:10.1002/hep.30651)
Supplement: Supplementary file 1 [file HEP-70-1377-s001.pdf]

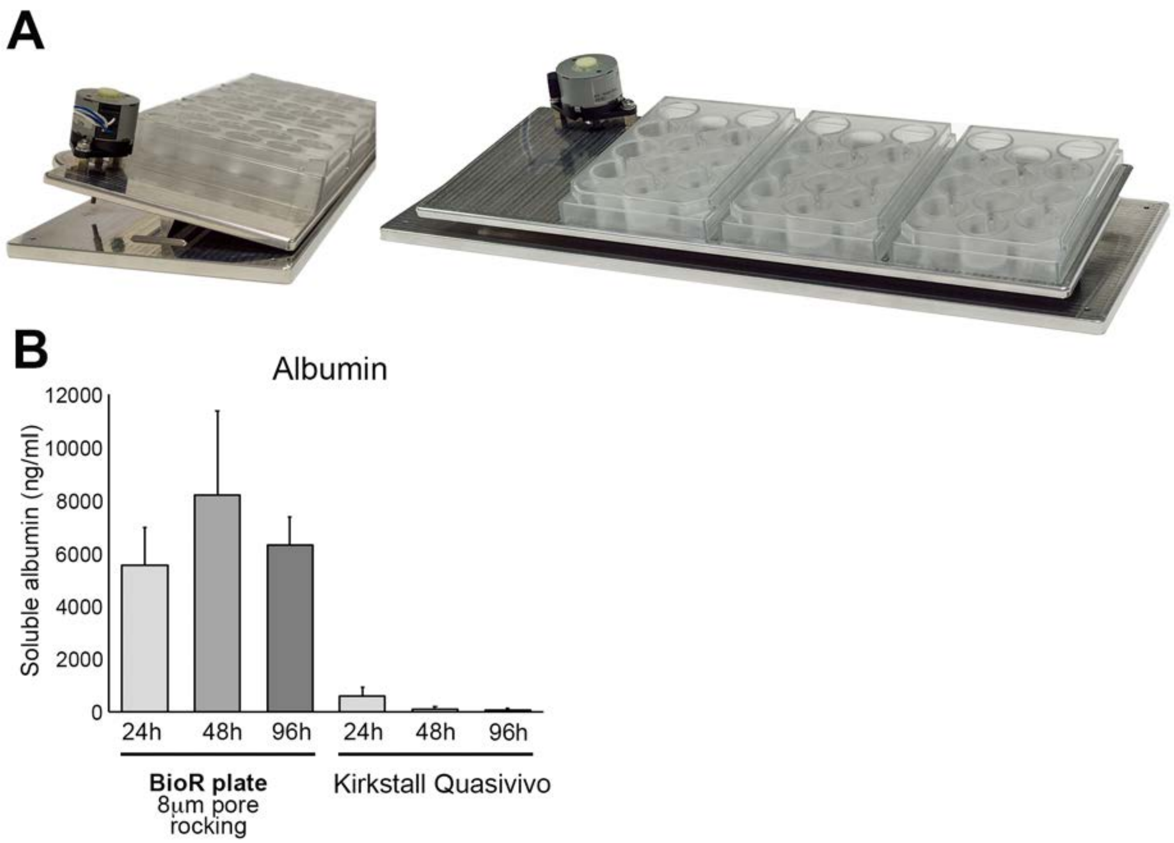

Supplementary figure 1- Bioreactor platform and culture plate.

(**A**) Photographs show the bioreactor rocker platform and BioR plate. (**B**) Graph showing media albumin levels in PCLS cultured in a Quasivivo circuit (unidirectional flow) or the BioR plate (bidirectional flow).

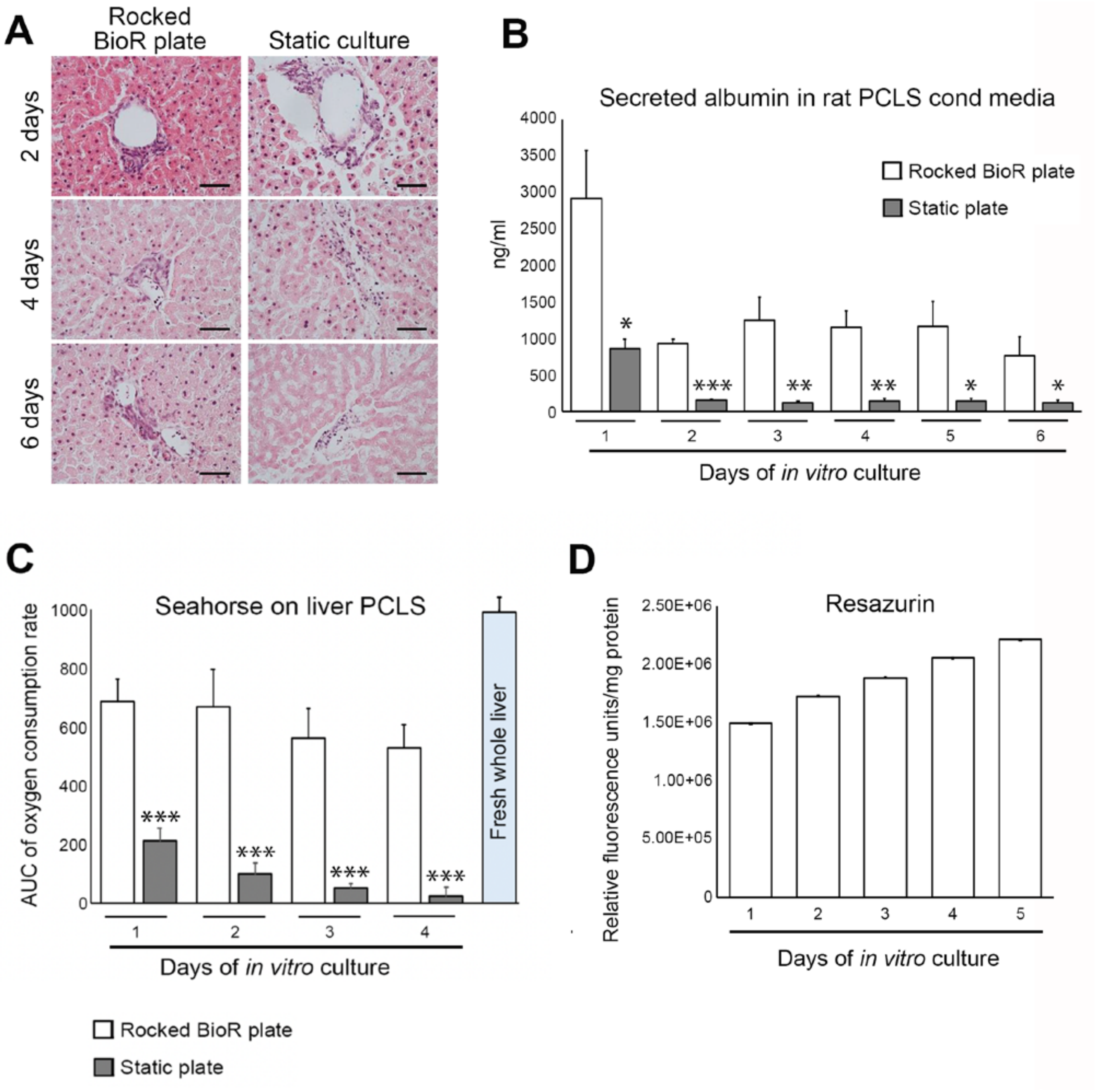

Supplementary figure 2- Bioreactor cultured PCLS have an extended healthy lifespan compared to static transwell cultured PCLS. (**A**) Representative H&E images show 2, 4 and 6-day bioreactor and static cultured rat PCLS. Scale bars equal 200 µm. (**B**) Graph shows average levels of albumin (ng/ml) released from static and bioreactor cultured rat PCLS into the culture media. (**C**) Graph shows metabolic activity measured by Seahorse assay expressed as area under the curve for oxygen consumption rate (AUC for OCR) in static and bioreactor cultured mouse PCLS. (**D**) Graph shows metabolic activity measured by Resazurin assay in static and bioreactor cultured mouse PCLS. Data represents mean ± SEM in n=4 independent slice experiments. * P <0.05, ** P <0.01 and *** P <0.001.

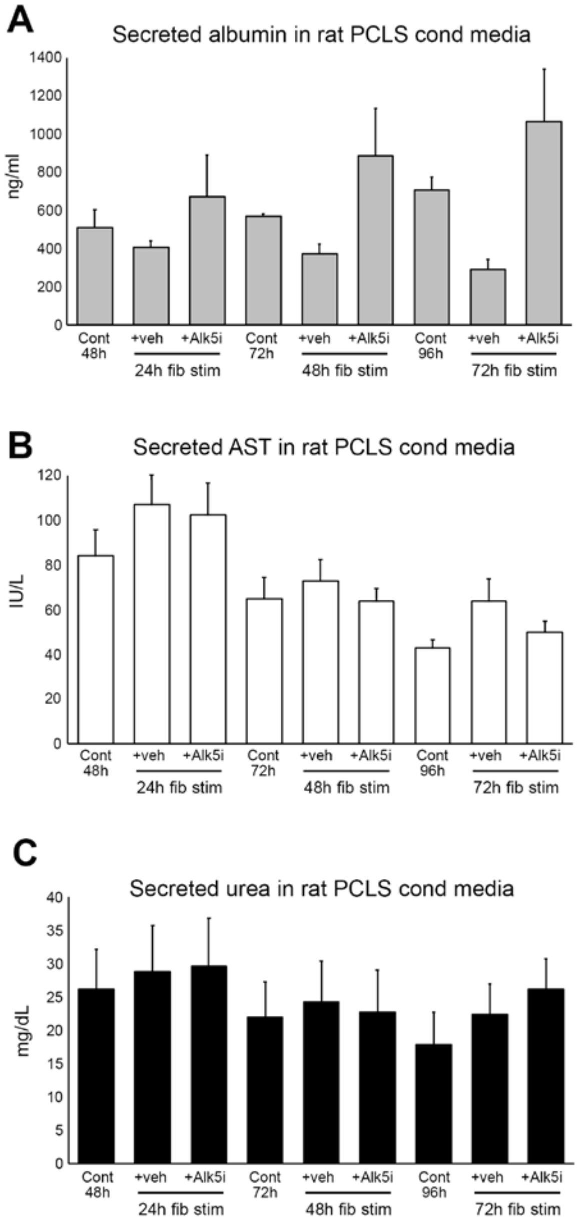

Supplementary figure 3- Viability and function of rat liver slices are not affected by profibrotic stimuli or an Alk5 inhibitor.

(**A-C**) Graphs show albumin (ng/ml), aspartate aminotransferase (IU/L) and Urea (mg/dL) levels in the culture media of bioreactor cultured rat PCLS after 24 rest and then 24h-72h culture ± fib stim (3ng/ml TGFβ1 and 50ng/ml PDGFββ) ± Alk5i. All data are presented as mean ± SEM in n=4 independent slice experiments.

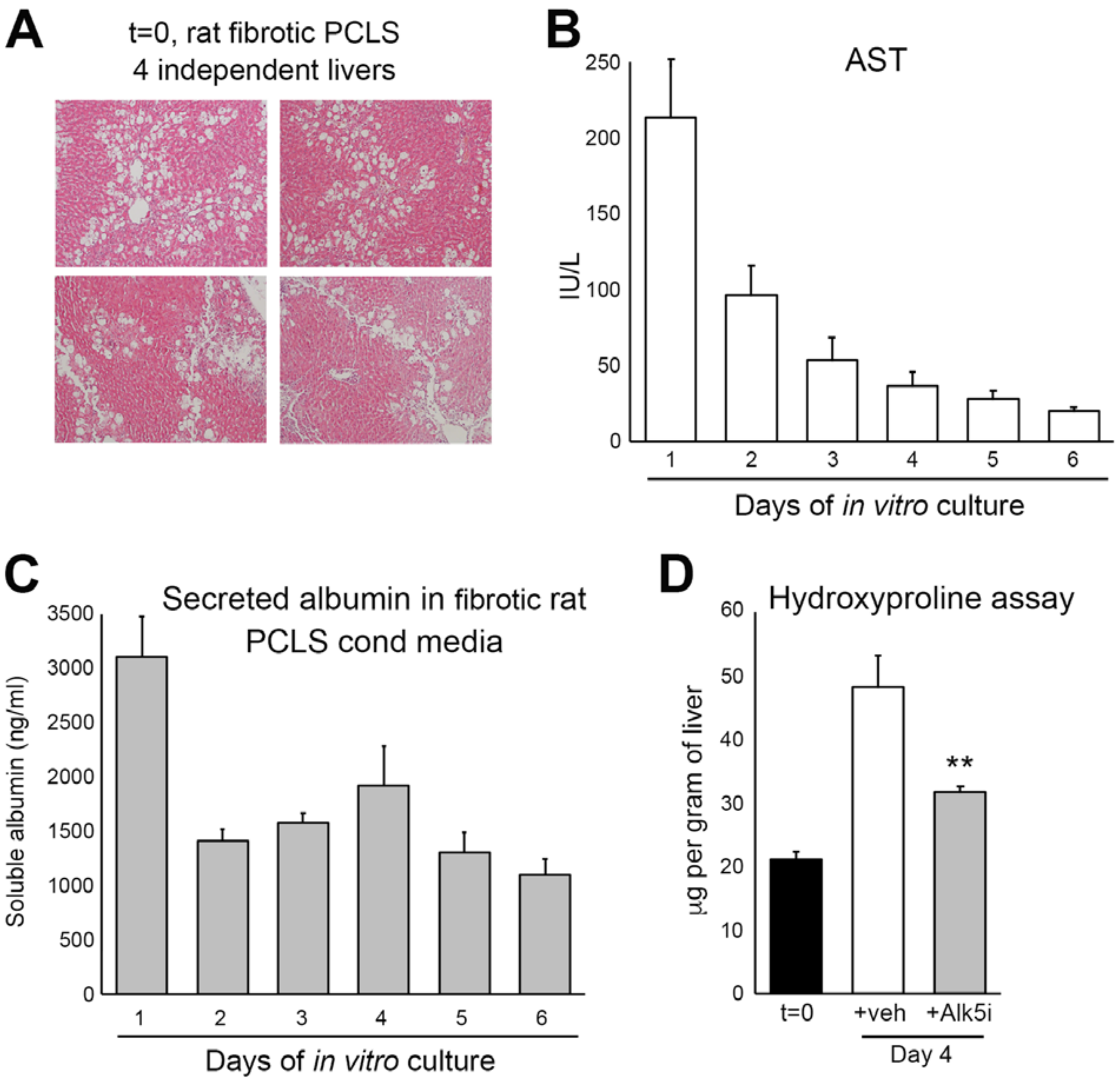

Supplementary figure 4- Functional characterisation of fibrotic rat liver slices.

(**A**) Representative 100x images of H&E stained sections from n=4 independent t=0 fibrotic rat PCLS. (**B-C**) Graphs show aspartate aminotransferase (IU/L) and albumin (ng/ml) levels in the culture media of fibrotic rat PCLS bioreactor cultured for 6 days. All data are presented as mean ± SEM in n=4 independent slice experiments. (**D**) Graph shows hydroxyproline in 4-day bioreactor-cultured fPCLS ± 10ng/ml Alk5i. Data are mean ± SEM in n=3 independent slice experiments. P values were calculated using an Anova with Tukey's multiple comparisons test (** P <0.01).

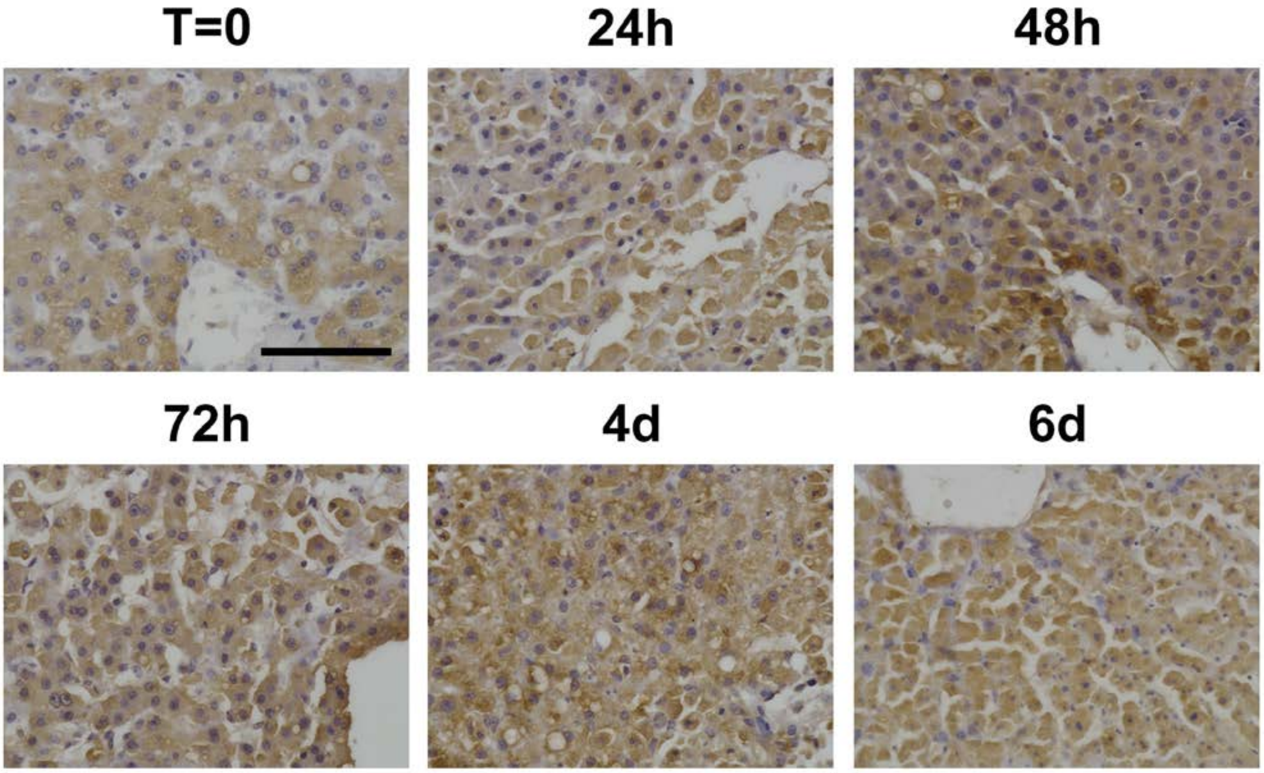

Supplementary figure 5 – Expression of CYP1A2 is retained in human PCLS over a 6-day culture period

Representative images show CYP1A2 positively stained hepatocytes at 400x magnification in T=0 and day 1, 2, 3, 4, and 6 cultured human PCLS.

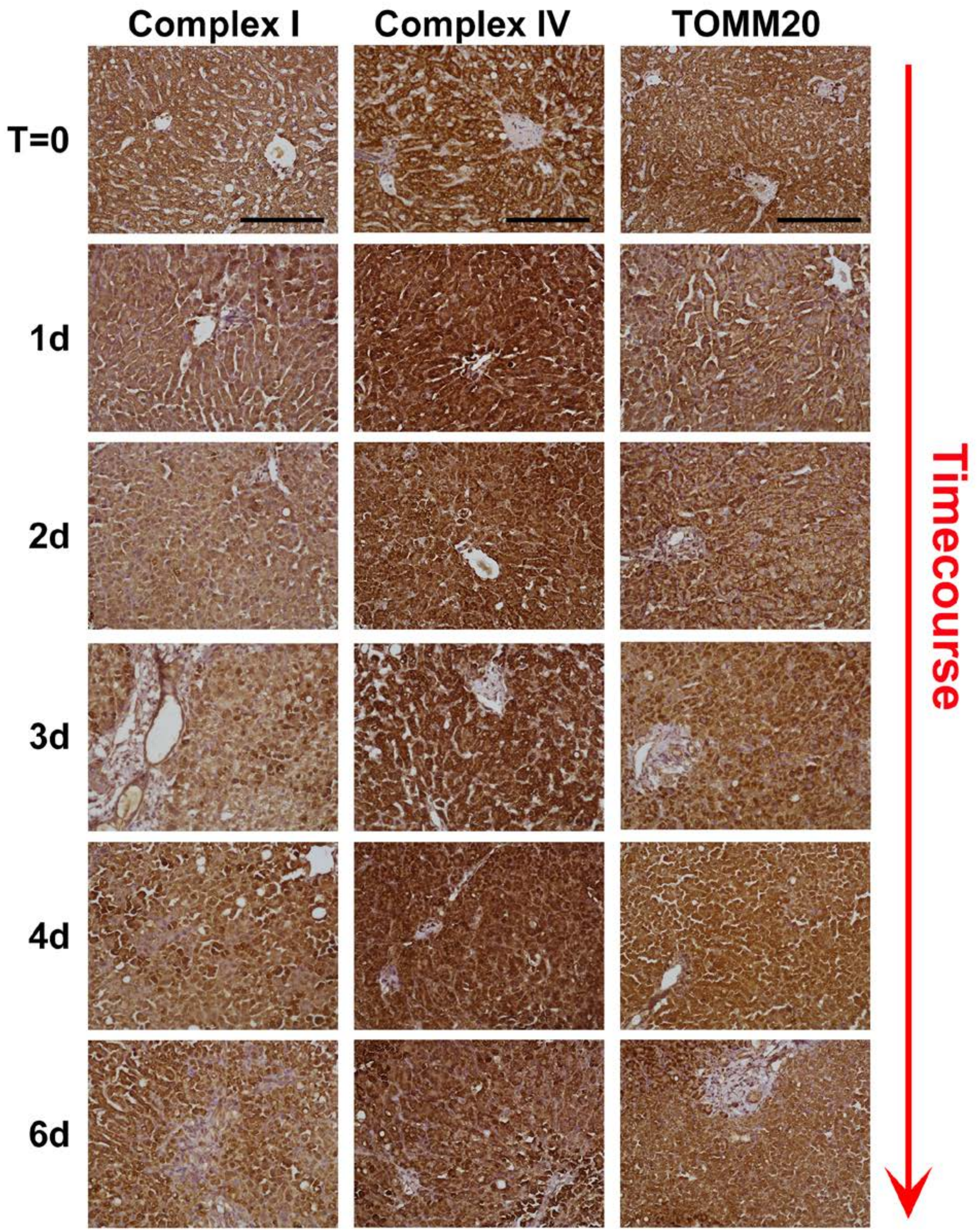

Supplementary figure 6- Expression of mitochondrial markers is retained in human PCLS over a 6-day culture period. Representative images show mitochondrial respiratory chain complexes I (NDUFB8) and complex IV (MTCO1) and the mitochondrial mass marker TOMM20 at 200x magnification in T=0 and day 1, 2, 3, 4, and 6 cultured human PCLS.

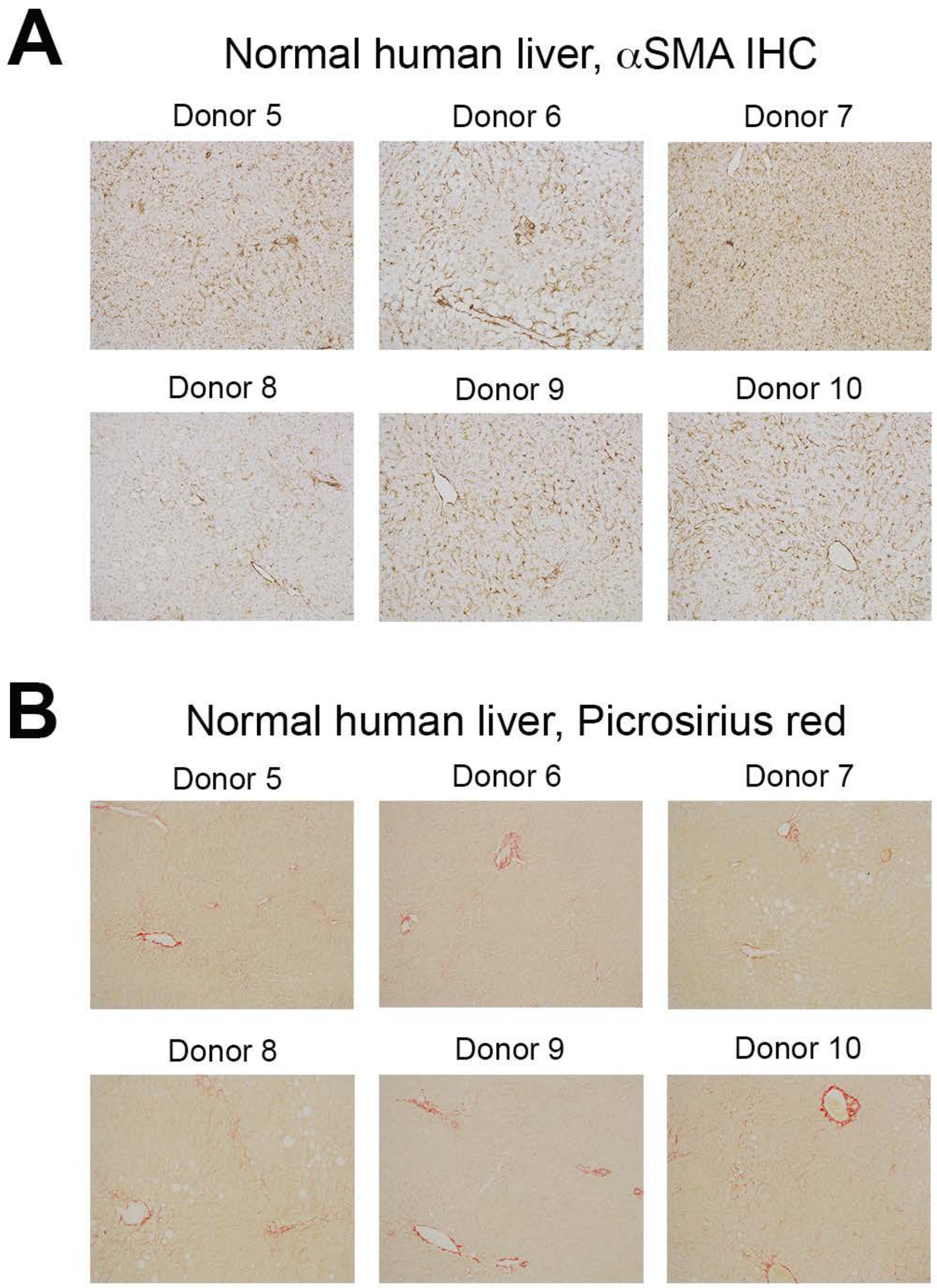

Supplementary figure 7- Picrosirius red and αSMA staining in normal human liver tissue

(**A**) Representative 100x images of αSMA and Picrosirius red stained sections from unsliced normal human liver tissue from the normal margin of a colorectal cancer resection. Images represent six different donor livers.

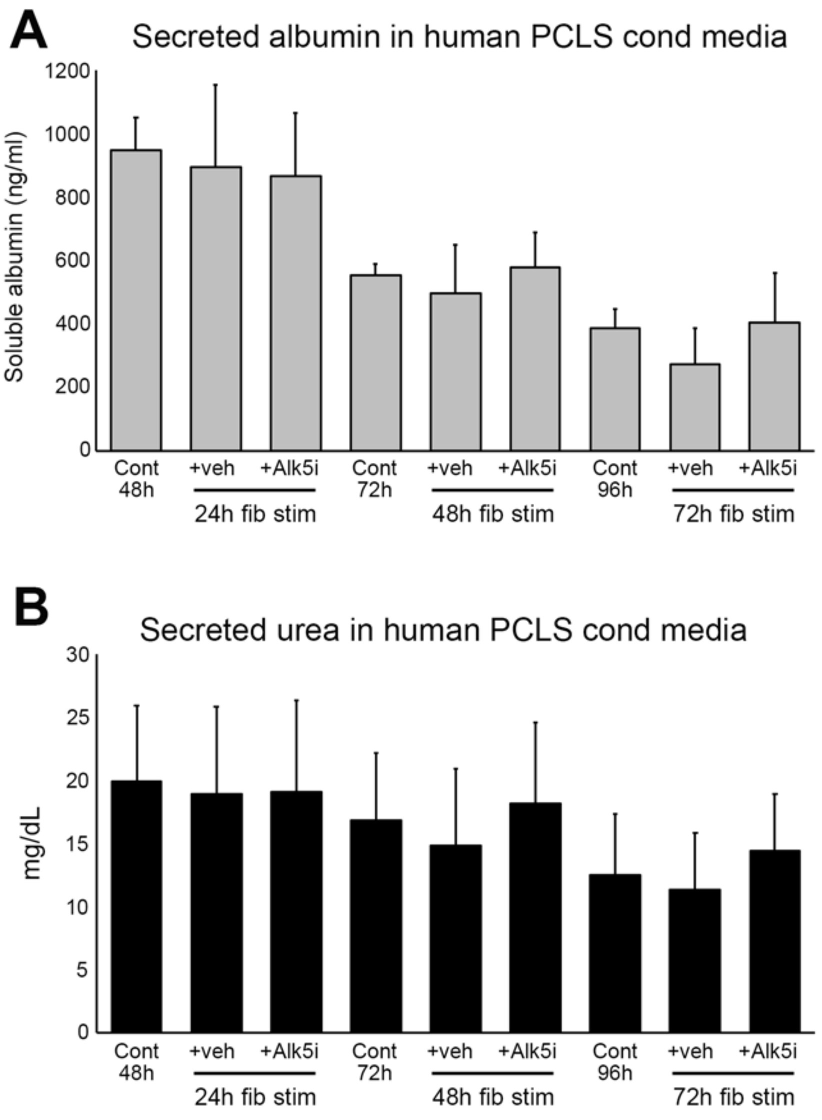

Supplementary figure 8 - Functional and histological characterisation of human liver slice treated with profibrotic stimuli

(**A-B**) Graph show albumin (ng/ml) and urea (mg/dL) levels in the culture media of bioreactor cultured human PCLS stimulated ± fib stim ± Alk5i. All data are presented as mean ± SEM in n=4 independent slice experiments.

Supplemental table 1: Rat and Human primer sequences.

Gene	Primer sequence
Rat GAPDH	Forward 5’-GCAAGAGAGAGGCCCTCAG-3’
	Reverse 5’-TGTGAGGGAGATGCTCAGTG-3’
Rat Collagen 1A1	Forward 5’-TTCACCTACAGCACGCTTGTG-3’
	Reverse 5’-GATGACTGTCTTGCCCCAAGTT-3’
Rat TIMP1	Forward 5’-TCTGGCATCCTCTTGGTTGCT-3’
	Reverse 5’-TAACCAGGTCCGAGTTGCAG-3’
Rat α-SMA	Forward 5’-CGAAGCGCAGAGCAAGAGA-3’
	Reverse 5’-CATGTCGTCCCAGTTGGTGAT-3’
Human GAPDH	Forward 5’-GTCAGTGGTGGACCTGACCT-3’
	Reverse 5’-TGAGCTTGACAAAGTGGTCG-3’
Human Collagen 1A1	Forward 5’-CAAGAGGAAGGCCAAGTCGAGG-3’
	Reverse 5’-CGTTGTCGCAGACGCAGAT-3’
Human TIMP1	Forward 5’-ACTTCCACAGGTCCACAAC-3’
	Reverse 5’-CATTCCTCACAGCCAACAGT-3’
Human α-SMA	Forward 5’-GCGTGGCTATTCCTTCGTTACT-3’
	Reverse 5’-CCGATGAAGGATGGCTGGAACA-3’

Supplementary methods

Histology/immunohistochemistry

5-µm-thick formalin-fixed, paraffin-embedded liver sections were processed for haematoxylin and eosin (H&E), Picrosirius Red and αSMA staining (1). Immunohistochemical staining for CD31, CK19 and CD68 were performed using routine methods at the pathology department, Royal Victoria Infirmary. CYP1a2 (Ab22717, Abcam, Cambridge, UK) immunohistochemical staining, briefly, slides were dewaxed, rehydrated and endogenous peroxidase activity was blocked using 0.6% hydrogen peroxide/methanol solution. Antigen retrieval was performed using antigen unmasking solution (H-3301, Vector laboratories, Peterborough, UK). Endogenous avidin and biotin was blocked for 20 minutes using an Avidin/Biotin Blocking Kit (SP-2001, Vector Laboratories, Peterborough, UK) and using 20% swine serum (C15SB, Bio-Rad, UK) for 30 minutes and then the primary antibody was added overnight at 4ºC. The following day slides were washed and incubated with biotinylated rabbit anti-mouse 1:200 (E0354 Dako, Ely, UK) for 1 hour and then washed and incubated with Vectastain Elite ABC HRP Reagent for 1 hour (PK-7100, Vector Laboratories, Peterborough, UK). Staining was visualised using DAB peroxidase substrate kit (SK-4100, Vector Laboratories, Peterborough, UK) prior to counterstain with mayers haematoxylin. Slides were then dehydrated and mounted using Pertex Mounted Medium (SEA-0100-00A, Cell Path, Newtown, UK). Immunohistochemical staining for mitochondrial submits/mass markers was preform as previously described (2) using the following antibodies; Complex I NDUFB8 1:50, Complex IV MTCO1 1:400, TOMM20 1:200 (All antibodies were purchased from Abcam)) in conjunction with a polymer detection system (Menarini Diagnostics). Images were acquired at x100 or x200 magnification using a Nikon ECLIPSE Ni-U microscope (NIS-Elements Br, Nikon, Kingston upon Thames, UK). The percentage Picrosirius red stained area was measured in fifteen-x200 magnification fields in Picrosirius red stained slides using Nikon Elements Imaging Software (NIS-Elements Br, Nikon, Kingston upon Thames, UK). The mean area was then calculated per slide.

Enzyme-linked immunosorbent assay

ELISA quantifications for rat COL1a1 (LS-F11152, LSBio, Nottingham, UK), human (E88-129) and rat (E110-125) albumin (Bethyl laboratories, Cambridge, UK), human fibronectin (DY1918-05, R&D systems, Abingdon, UK) and human hyaluronic acid (DY2089, R&D systems, Abingdon, UK) were performed as per manufacturer's instructions.

Luminex assay

Quantifications for human TIMP1 (milliplex HTMP2MAG-54K), MMP1, MMP7 and MMP10 (milliplex HTMP2MAG-55K Thermofisher Scientific, Cramlington, UK) were performed as per manufacturer's instructions.

Colorimetric assay

Aspartate aminotransferase (AST) levels were measured by the Clinical pathology department, Royal Victoria infirmary, Newcastle-Upon-Tyne, UK.

Hydroxyproline assay

Two PCLS were weighed and digested with 1ml of 6N HCl to measure the quantity of hydroxyproline as previously described(3).

RNA isolation, cDNA synthesis and PCR

2-4 PCLS per condition were placed in QIAzol, disrupted in a Qiagen Tissue Lyser II and passed through a Qiashredder (Qiagen, Manchester, UK). Chloroform was added, the sample vortexed and centrifuged at 12,000g for 15 minutes. The aqueous layer was collected and added to 70% ethanol. Total RNA was purified using the RNeasy Micro Kit (Qiagen, Manchester, UK). 1µg RNA was treated with 1µl DNase (Promega, Southampton, UK) for 30 minutes at 37°C and first strand cDNA produced *via* incubation with random hexamer primer (p(dN)6) and 100 units MMLV reverse transcriptase as previously described (4). Real-time

polymerase chain reaction was performed with SYBR Green JumpStart Taq ready mix (Sigma, Poole, UK) as per manufacturer's instructions using primers listed in supplemental table 1. Relative level of transcriptional difference (RLTD) was calculated using the $2^{\Delta\Delta Ct}$ method.

PCLS Respirometry

Bioenergetics of PCLS was determined using a Seahorse respirometer as previously described (5). Briefly, small pieces (6-10mg) of mouse liver or PCLS were rinsed with Krebs-Henseleit buffer (KHB) (111 mM NaCl, 4.7 mM KCl, 2 mM $MgSO_4$, 1.2 mM $Na2HPO_4$, 0.5 mM carnitine, 2.5 mM glucose and 10mM sodium pyruvate) and then transferred to individual wells of a XF24 plate. Liver tissue/PCLS were stabilized from excessive movement by islet capture screens (Seahorse Bioscience), and 450 mL KHB was added to each well. Digitonin was then added to enhance plasma membrane permeability. Basal oxygen consumption rates (OCR) were determined at 37°C according to the following plan: Basal readings recorded every 2 minutes for 5 readings, followed by exposure to digitonin. Subsequent readings were recorded after 2 minutes mixing and 2 minutes rest. Basal OCR values were normalized to individual tissue weights.

Statistical Analysis

Data are mean ± standard error of the mean (SEM), where *, **, *** and **** signify P values of <0.05, <0.01, <0.001 and <0.0001 respectively. P values were calculated using GraphPad7 prism software and an ANOVA with Tukey Post-hoc test or unpaired T-test.

References

1. Oakley F, Mann J, Nailard S, Smart DE, Mungalsingh N, Constandinou C, Ali S, et al. Nuclear factor-kappaB1 (p50) limits the inflammatory and fibrogenic responses to chronic injury. Am J Pathol 2005;166:695-708.
2. Greaves LC, Barron MJ, Plusa S, Kirkwood TB, Mathers JC, Taylor RW, Turnbull DM. Defects in multiple complexes of the respiratory chain are present in ageing human colonic crypts. Exp Gerontol 2010;45:573-579.
3. Wynn TA, Barron L, Thompson RW, Madala SK, Wilson MS, Cheever AW, Ramalingam T. Quantitative assessment of macrophage functions in repair and fibrosis. Curr Protoc Immunol 2011;Chapter 14:Unit14 22.

4. Oakley F, Meso M, Iredale JP, Green K, Marek CJ, Zhou X, May MJ, et al. Inhibition of inhibitor of kappaB kinases stimulates hepatic stellate cell apoptosis and accelerated recovery from rat liver fibrosis. Gastroenterology 2005;128:108-120.
5. Martinez-Lopez N, Garcia-Macia M, Sahu S, Athonvarangkul D, Liebling E, Merlo P, Cecconi F, et al. Autophagy in the CNS and Periphery Coordinate Lipophagy and Lipolysis in the Brown Adipose Tissue and Liver. Cell Metab 2016;23:113-127.
